# The Status of Bile Acids and Farnesoid X Receptor in Brain and Liver of Rats with Thioacetamide-Induced Acute Liver Failure

**DOI:** 10.3390/ijms21207750

**Published:** 2020-10-20

**Authors:** Anna Maria Czarnecka, Krzysztof Milewski, Jan Albrecht, Magdalena Zielińska

**Affiliations:** Department of Neurotoxicology, Mossakowski Medical Research Centre, Polish Academy of Sciences, 5 Pawińskiego Street, 02-106 Warsaw, Poland; kmilewski@imdik.pan.pl (K.M.); jalbrecht@imdik.pan.pl (J.A.)

**Keywords:** acute liver failure, bile acids, farnesoid X receptor, immunocytochemistry, small heterodimer partner, thioacetamide

## Abstract

Acute liver failure (ALF) leads to neurological symptoms defined as hepatic encephalopathy (HE). Although accumulation of ammonia and neuroinflammation are generally accepted as main contributors to HE pathomechanism, a buildup of bile acids (BA) in the blood is a frequent component of liver injury in HE patients. Recent studies have identified the nuclear farnesoid X receptor (FXR) acting via small heterodimer partner (SHP) as a mediator of BA-induced effects in the brain of ALF animals. The present study investigated the status of the BA–FXR axis in the brain and the liver, including selective changes in pertinent genes in thioacetamide (TAA)-induced ALF in Sprague–Dawley rats. FXR was found in rat neurons, confirming earlier reports for mouse and human brain. BA accumulated in blood but not in the brain tissue. Expression of mRNAs coding for *Fxr* and *Shp* was reduced in the hippocampus and of *Fxr* mRNA also in the cerebellum. Changes in *Fxr* mRNA levels were not followed by changes in FXR protein. The results leave open the possibility that mobilization of the BA–FXR axis in the brain may not be necessarily pathognomonic to HE but may depend upon ALF-related confounding factors.

## 1. Introduction

Hepatic encephalopathy (HE) is a complex neurological syndrome associated with acute or chronic liver failure (ALF or CLF). ALF, which results from fulminant action of hepatotoxins, is a cause of type A HE, a condition invariably manifested by rapid progression of neurotransmission imbalance resulting in pre-coma to coma and by an evolution of brain edema, which is a major cause of death [1]. While ammonia and inflammatory mediators are well documented and probably the major contributors to the neurological manifestations of ALF [2,3], they do not exhaust the list of potential pathogenic factors in HE. Ever since the appearance of a seminal report by Roger Williams group in 1977 [4], numerous studies have emphasized accumulation in the blood of bile acids (BA), which are synthesized primarily in the liver from cholesterol, as a consistent marker of liver dysfunction primarily in cholestatic liver disease and in HE as well [5,6,7]. Elevation of plasma BA during liver failure leads to the excessive rise in cerebral BA levels in humans and rodents [4,8,9,10] most likely arising in there from the systemic circulation through disrupted brain–blood barrier (BBB) [11]. The presence of the alternative pathway of BA synthesis in the brain [12], along with the widespread distribution of several types of BA receptors in the human and rodent brain [8,13,14] prompts interests of BA signaling in the context of HE-associated neurological dysfunctions.

There is a growing consensus that both the feedback regulation of BA synthesis and metabolic control by BA of numerous intracellular metabolic pathways are coordinated by the activity of the farnesoid X receptor (FXR), a member of the nuclear receptor superfamily. After BA binding to FXR, it induces the expression of small heterodimer partner (SHP) [15,16]. FXR regulates transcription of genes involved in BA synthesis, absorption, uptake, and transport maintaining BA homeostasis [12,14]. Advancement of BA from the peripheral marker of liver damage to a potential carrier of HE came with the demonstration that in mice with azoxymethane (AOM)-induced ALF, BA detected in the brain, most likely driven by the intracerebral accumulation of cholesterol [17] activate FXR and contribute to HE symptoms [8].

The presence of FXR in the central nervous system, long contested in the past [18], was confirmed in a few studies in the cerebral cortex [8] and hippocampus [14]. The ambiguous response FXR in the cerebral cortex of mice with AOM-induced ALF [8,10] turned our attention to FXR signaling also in other brain areas affected in HE, particularly the cerebellum [19,20,21] and the hippocampus [22]. Of note in this context, many manifestations of HE are of cerebellar origin and occur early, when the cerebral cortex appears unaffected as yet [19,21].

Thioacetamide (TAA) treatment has been reported to downregulate FXR in the liver [23,24] and the administration of FXR agonists has led to the improvement of morphology, biochemistry, and function in the liver of TAA-treated mice [25,26,27]. It thus appeared of interest to find out whether and to what degree, the response of FXR to BA in the liver is reflected in the brain.

Therefore, we focused our research on the FXR and SHP expression in three selected brain structures, the cerebral cortex, cerebellum, and the hippocampus in the TAA-induced rat model of the ALF. Concomitantly, the analysis was conducted in the liver, a reference tissue for FXR-related changes. We have chosen to use TAA to induce ALF in rats, since it is a well-established and widely recommended model of type A HE [28] with rather a preserved BBB integrity [29,30]. The specific aims of this study were to identify the type of brain cells expressing FXR, perform quantitative analysis of FXR expression profile in the brain regions affected in HE, and estimate the total BA concentration in the plasma and brain tissue homogenate.

## 2. Results

### 2.1. The Pattern of Immunoreactivity for Farnesoid X Receptor

#### 2.1.1. Brain (Cerebral Cortex, Hippocampus, Cerebellum)

**Figure 1:**
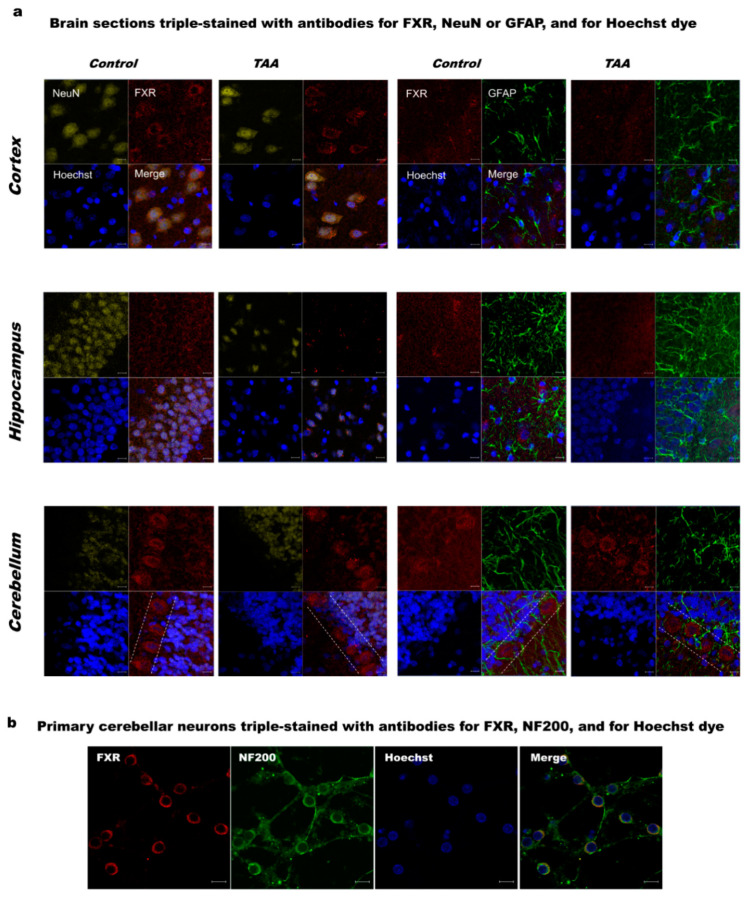
(**a**) Immunolabelling of farnesoid X receptor, FXR (red) in rat cerebral cortex, hippocampus, and cerebellum of control and thioacetamide (TAA) rats. Neurons were positively immunostained against the neuronal marker NeuN (yellow). Astrocytes were identified by immunostaining against the glial fibrillary acidic protein (GFAP, green). Blue Hoechst stain indicates the location of cell nuclei. Dashed lines indicate the Purkinje layer. Scale bars = 10 μm; (**b**) immunolabelling of FXR in the primary cultures of rat cerebellar neurons. Neurons were positively immunostained against the neuronal marker neurofilament 200 (NF200, green). Blue Hoechst stain indicates the location of cell nuclei. Scale bars = 10 μm.

Immunocytochemical labeling of brain slice sections with the FXR antibody revealed dominant localization of FXR protein in neurons. FXR was distributed both in the cytoplasm and nucleus with a characteristic pattern around the nucleus. FXR staining was most prominent in the area of the Purkinje layer of the cerebellum (Figure 1a). Immunofluorescence staining showed no specific changes in the FXR expression pattern between control and TAA brains (Figure 1a).

#### 2.1.2. Primary Cultures of Cerebellar Neurons

Since cerebellar neurons exhibited the strongest FXR immunoreactivity among the brain areas tested, cerebellar granule neurons were chosen for primary neuronal cultures. The FXR protein showed predominantly nuclear localization (Figure 1b).

### 2.2. Total Bile Acids Concentration

**Figure 2:**
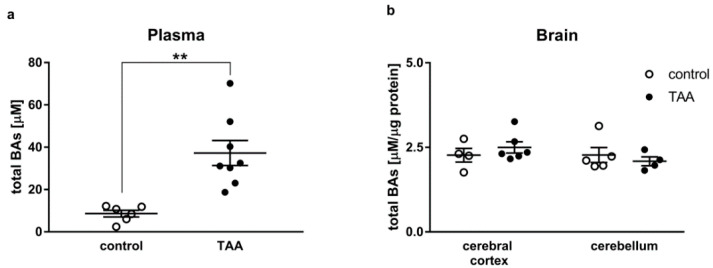
Total bile acids concentration in the plasma (**a**) and brain tissue homogenates (cerebral cortex and cerebellum) (**b**) of control and TAA rats. Black circles represent the TAA-treated group, whereas empty circles denote the control group. All data are presented as an overlaying scatter-plot of single data points ± SEM, *n* = 4–8. ** *p* < 0.01 compared with control.

Total BA concentration increased approx. 430% in the plasma of ALF rats (*p* < 0.01, Figure 2a). There were no differences in the BA contents of the cerebral cortex and cerebellum homogenates between control and TAA rats (Figure 2b).

### 2.3. Real-Time qRT-PCR and Western Blot Analysis of the Expression of FXR and SHP

#### 2.3.1. Liver

**Figure 3:**
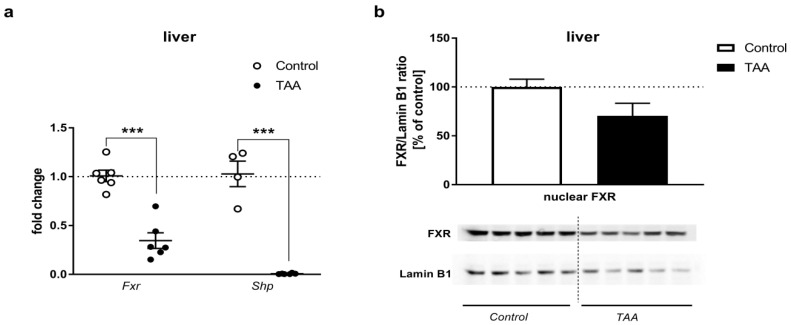
(**a**) *Fxr* and *Shp* mRNA expression in the liver of control and TAA rats. Black circles represent the TAA-treated group, whereas empty circles denote the control group. All data are presented as an overlaying scatter-plot of single data points ± SEM, *n* = 4–6. ***p < 0.001 compared with control; (**b**) the relative protein level of FXR (55 kDa) in the nuclear fraction of liver homogenate of control and TAA rats. Lamin B1 (66 kDa) was used as an internal control. Results are presented as the mean ± SEM, *n* = 5.

ALF resulted in a downregulation of *Fxr* (approx. of 65%, *p* < 0.001) and *Shp* (approx. of 99%, *p* < 0.01) mRNA expression (Figure 3a). The cytoplasmic fraction of the liver showed no detectable expression of FXR protein (data not shown). FXR protein expression in the liver was not significantly affected by TAA treatment as measured in the nuclear fraction of tissue homogenates, however, a tendency towards down-regulation can be observed, *p* = 0.083 (Figure 3b).

#### 2.3.2. Brain

**Figure 4:**
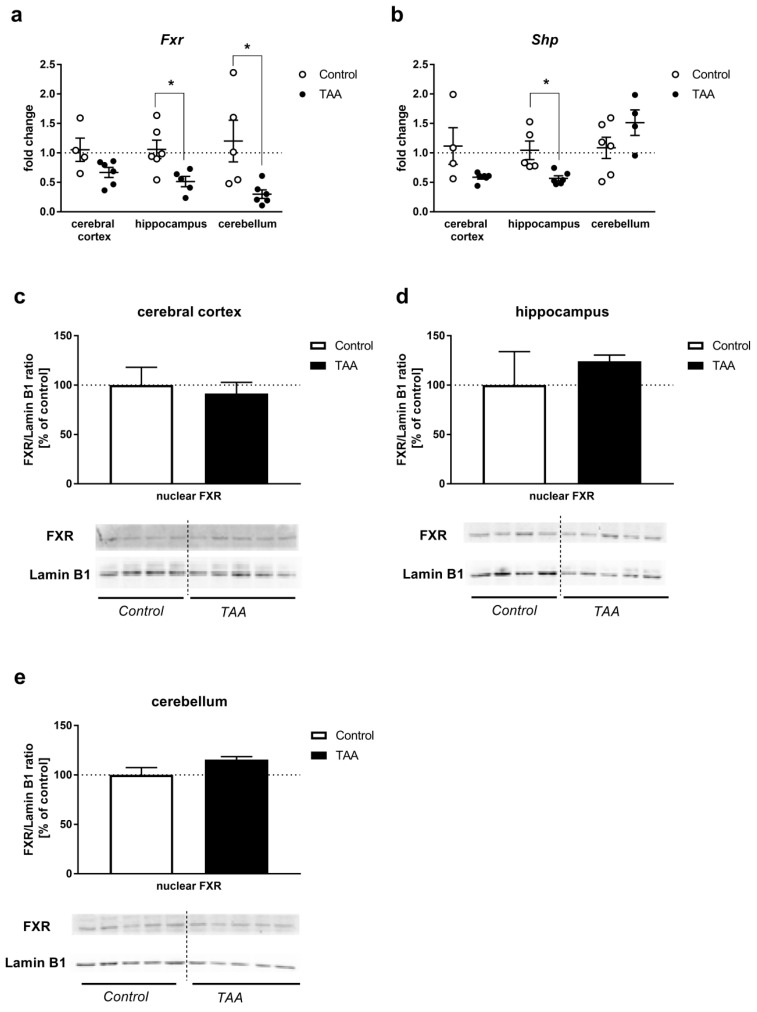
*Fxr* and *Shp* mRNA expression in the cerebral cortex, hippocampus, and cerebellum of control and TAA rats (**a**,**b**). Black circles represent the TAA-treated group, whereas empty circles denote the control group. All data are presented as an overlaying scatter-plot of single data points ± SEM, *n* = 4–6. * *p* < 0.05 compared with control; the relative protein level of FXR (55 kDa) in the nuclear fraction of cerebral cortex (**c**), hippocampus (**d**), cerebellum (**e**), homogenates of control and TAA rats. Lamin B1 (66 kDa) was used as an internal control. Results are presented as the mean ± SEM, *n* = 4–5.

*Fxr* mRNA level was decreased (hippocampus and cerebellum, *p* < 0.05) or unaltered (cerebral cortex) by TAA (Figure 4a). *Shp* mRNA level was decreased (hippocampus, *p* < 0.05) or unaltered (cerebral cortex, cerebellum, Figure 4b). Relative gene expression levels both for *Fxr* and *Shp* were approx. 1000 times lower in all analyzed brain structures when compared to the liver (data not shown). FXR protein expression remained unaffected by TAA treatment in all analyzed brain structures as measured in the nuclear fraction of tissue homogenates (Figure 4c–e).

## 3. Discussion

This study documented changes in *Fxr/Shp* genes expression in selected brain regions upon ALF induction by TAA, even though the increase in BA in the blood was not accompanied by its accumulation in the brain. Another observation of the study shows, to our knowledge for the first time, a predominant localization of FXR in the cerebellum among the studied brain areas, relatively most abundant in the cerebellar Purkinje layer. However, this result requires further quantitative and comparative validation in relation to other locations and types of neurons.

Recent reports [8,10,17] implicate BA signaling as a new factor in the pathogenesis of HE. It is suggested that total BA in the serum could serve as an additional marker for risk stratification in patients with cirrhosis [31]. Of note, in the pioneering study of Bron et al. [4], increased blood BA was invariably recorded in comatose patients with ALF elicited by different causes (paracetamol ingestion, viral hepatitis, and hepatotoxicity of ill-defined origin), but cerebrospinal fluid (CSF) BA levels did in no case correlate with brain edema, the primary cause of death. The latter study advocates caution when implicating BA in the individual cases or manifestations of HE.

Although a circulating level of total BA substantially increased in the present study, their concentration measured in brain tissues remained unaffected most likely due to the preserved BBB integrity. In contrast to the substantial BBB breakdown accompanying AOM-induced ALF [32,33,34,35,36], a lack of discernible BBB damage has been consistently reported in the TAA rat model [37,38,39,40]. The relatively high vulnerability of BBB to AOM as compared to TAA or one other ALF modeling hepatotoxin, galactosamine, was demonstrated in an astroglia/brain endothelial co-culture in vitro [29]. In line with the above, only AOM, but not TAA, intoxication resulted in a substantial increase in BA in the cerebral cortex [30].

The question of whether BA are capable of exerting any relevant effects via FXR-mediated cerebral signaling will have to be resolved by a more sophisticated LC/MS analysis of the different BA pools, since BA profile may substantially vary in the course of HE.

Interestingly, the concurrence of decreased expression of *Fxr* mRNA with the unaltered expression of FXR protein in brain regions is a mirror reflection of the responses occurring in the liver. The striking disparity between the drop of *Fxr* mRNA and lack of change in FXR protein content needs to be elucidated. In most simple terms, the result may manifest a delay in the onset of protein loss vs. the precedent loss of mRNA. The loss of *Fxr* mRNA may have occurred due to one or a combination of the following events: inhibition of transcription; RNA methylation [41]; impaired RNA stability, possibly reflecting regional differences in the redox status [42]. Since the clinical manifestation of ALF is obviously a dynamic process, the documentation of the changes in FXR expression in the brain in the time course of HE progression would be a determining factor. The observation of decreased *Fxr* mRNA in the liver confirms the earlier findings (see introductory paragraphs). Apart from the downregulation of *Fxr* and *Shp* expression in two of the examined brain areas, an altered spectrum of individual BA in the TAA-affected brain may likewise contribute to cerebral FXR signaling. This is the case in the brains of bile-duct-ligated rats, where notwithstanding the lower total amount of BA than in controls, the BA profile suggested overrepresentation of its toxic constituents [43]. The exact composition of blood and brain BA pools in TAA-treated rats and its potential impact of FXR signaling remain to be investigated further.

The very first demonstration of FXR signaling, as a component of acute HE pathogenesis implicated in the neurological decline, was documented in a mouse AOM model, where activation of the BA–FXR axis was triggered by BA flux from the liver to the brain [8]. The present results do not support the increased brain *Fxr/Shp* mRNA and protein expression, nor a beneficial effect of FXR silencing previously reported in AOM mice [8,17]. The question of whether and in what degree changes in brain FXR signaling are pathognomonic to HE or depend upon ALF-related confounding factors remains open to further endeavors. However, the concomitantly presented data of liver-specific response in the TAA model are the advantage of our experimental protocol in which ALF-induced systemic BA caused a reduction in the expression of mRNAs coding for *Fxr* and *Shp*. Considering that only a part of the BA biosynthesis pathway is present in the brain [12], we presume that the main role of brain FXR activation is not feedback inhibition of BA synthesis but the control of various signaling pathways via binding to FXR response elements in different target genes. Clearly, precise identification of particular signaling pathway needs further study. Taking into account that the use of specific FXR agonist is suggested as a promising therapy in a broad spectrum of diseases, including hepatic failure, metabolic syndrome, diabetes, obesity, or vascular impairment occurring in liver cirrhosis [16,44,45,46], our data contribute to the idea that strengthening of FXR/SHP signaling may be beneficial in the treatment of the ALF.

We indicated neurons as the main brain cell type expressing FXR and observed a relatively high abundance of FXR in the cerebellar Purkinje layer. The status of the BA–FXR axis in the brain of rats with TAA-induced ALF included regional differences in *Fxr* and *Shp* expression. Importantly enough, the cerebral FXR/SHP alterations were consistent with those in the liver, despite that in contrast to the elevated BA in the serum, the concentration of brain BA remained unaltered. Experimental screening of the effects of natural and synthetic compounds for their effects on the BA–FXR/SHP axis is likely to offer the desired answers regarding their role in the progression of human HE. The generated answers may aid future attempts at designing treatment modalities.

## 4. Materials and Methods

### 4.1. The Acute Liver Failure Model

**Figure A1:**
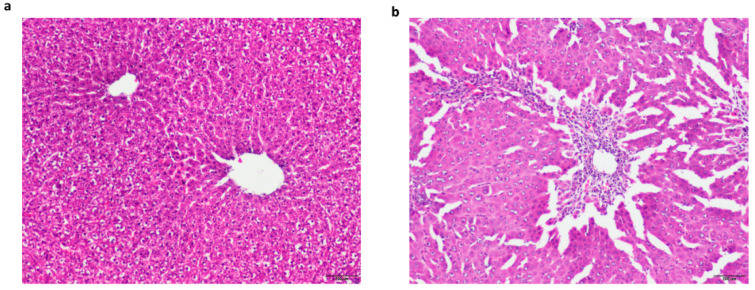
Light micrographs of hematoxylin-eosin stained liver sections from control (**a**) and TAA-treated (**b**) rats. The histology studies of the control liver showed the normal histological appearance of liver tissue with central vein and portal area (**a**), whereas TAA-treated liver section revealed widespread intracellular vacuolization, centrilobular bridging hepatocellular necrosis, and infiltration of inflammatory cells (**b**). Scale bars = 200 μm.

Male Sprague–Dawley rats of the initial body weight ~250 g from the outbred animal colony (Tac: Cmd: SD) were supplied by the Animal House of Mossakowski Medical Research Centre, Warsaw, Poland. Animals were kept in 2–3 in cages at temperature 22 °C under an artificial light/dark cycle (12/12 h), with access ad libitum to standard laboratory chow food and tap water. All procedures were carried out following the Directive 2010/63/EU and with approval of the 1st Local Ethics Committee for Animal Experimentation, Warsaw, Poland (the approval number 359/2017, 4 July 2017). Animals received three intraperitoneal (ip) injections of TAA (Sigma-Aldrich, Steinheim, Germany) (300 mg/kg of body weight) per day for three consecutive days at 24 h intervals. Control rats were injected ip with saline. Twenty-four hours after the third injection of TAA rats were decapitated, and liver, brain, and blood samples from all groups of animals were collected. Induction of the acute liver injury was confirmed by the analysis of biochemical markers of liver injury (plasma level of ammonia and activities of aspartate aminotransferase, alanine aminotransferase, and γ-glutamyl transpeptidase) (Table A1), as well as hematoxylin and eosin staining of liver sections derived from right lobes (block preparation in paraffin, section cutting 5–6 µm thick) (Figure A1). All efforts were made to reduce the number of animals used and their suffering. The study complies with the ARRIVE guidelines for reporting animal research.

### 4.2. Primary Neuronal Cultures

The cerebellar neurons were prepared from 7-day-old rat Wistar pups (12–18 g). Cerebellar granule cells were isolated and cultured according to the method described by [47] with modifications [48]. The isolated cerebella were placed in a 35 mm Petri dish, catted into slices, and incubated in Krebs ionic buffer containing 0.025% trypsin (Sigma Aldrich, Steinheim, Germany) and 0.05% DNase (Roche, Basel, Switzerland). Subsequently, a trypsin inhibitor was added; the cerebellar slices were collected via centrifugation, triturated, and re-centrifuged. The cells were suspended in basal medium eagle supplemented with 10% fetal calf serum, 25 mM KCl, penicillin/ streptomycin (Sigma Aldrich, Steinheim, Germany) and passed through a syringe with a thick needle. After centrifugation, the single-cell suspension was seeded onto poly-L-lysine-coated glass coverslips at a density of 125–250 × 10^3^ cells/well. Forty-eight hours after seeding, 1.2 mM glucose and 7.5 mM cytosine arabinofuranoside (Sigma Aldrich, Steinheim, Germany) were added to inhibit the proliferation of astrocytes and fibroblasts. Immunolabeling was performed on 7-day neurons.

### 4.3. Immunolabelling

Immunolabelling and the acquisition of images were performed following the procedure described previously [49]. The frozen tissue blocks from brain structures were sectioned using the cryotome into 25 µm thickness slices. Immunohistochemistry was performed on 4–6 sections of each examined brain regions of experimental (*n* = 3) and control animals (*n* = 3), and 3–4 fields of each section were evaluated. Cells or tissue slices were incubated with primary antibodies against FXR (1:250, 417200, Invitrogen, MA, USA) and glial fibrillary acidic protein (GFAP, 1:400, mouse monoclonal, MAB360, Merck, Darmstadt, Germany) or NF200 (1:400, N0142, Sigma-Aldrich, Steinheim, Germany) or NeuN (1:500, ab196584, Abcam, Cambridge, MA, USA) overnight at 4 °C. On a consecutive day, the cells or slice sections were exposed to goat anti-mouse IgG2a Alexa Fluor 546 and goat anti-mouse IgG1 Alexa Fluor 488-conjugated secondary antibodies (1:500, Invitrogen, Waltham, MA, USA). The cell nuclei were stained with Hoechst 33258 (Invitrogen, Waltham, MA, USA). To obtain the detailed images of the labeled cells, a confocal laser scanning microscope LSM 780 (Zeiss, Oberkochen, Germany) was used.

### 4.4. Real-Time PCR

RNA was extracted from flash-frozen tissue of the rat liver, rat brain cerebral cortex, hippocampus, cerebellum using TRI Reagent (Sigma-Aldrich, Steinheim, Germany). An amount of 2 µg was reverse transcribed using the high-capacity cDNA reverse transcriptase kit (Applied Biosystems, Foster City, CA, USA). Real-time PCR was performed in 96-well plates with the ABI 7500 apparatus (Applied Biosystems, Foster City, CA, USA). The mRNA expression was determined by Taqman Gene Expression Assay (Applied Biosystems, Foster City, CA, USA), using 1.5 μL of cDNA in a reaction of 10 μL. The assay IDs were Rn00572658_m1 for rat *Nr1h4* (*Fxr*), Rn00589173_m1 for *Nr0b2* (*Shp*), and Rn00589173_m1 for *β-actin*. The fold change in the gene expression was determined by the 2^-ΔΔCt^ method [50]. The relative gene expression levels of *FXR* mRNA were normalized to *β-actin*. Expression changes were calculated as a fold change relative to the control samples.

### 4.5. Nuclear Protein Isolation and Western Blot Analysis

Nuclear fraction was extracted from the liver and brain tissue using NE-PER Nuclear and Cytoplasmic Extraction Reagents (Thermo Fisher Scientific, Waltham, MA, USA) according to the manufacturer protocol. Protein concentration was estimated using the BCA kit (Thermo Fisher Scientific, Waltham, MA, USA).

The relative protein content was determined by Western blotting as previously described [51]. Amounts of 15 µg (liver) or 30 µg (brain structures) of nuclear proteins, extracted from tissues of control and TAA rats, were used for analyses. Nitrocellulose membranes were incubated overnight with an anti-FXR antibody (1:1000; mouse monoclonal IgG2a, 417200, Thermo Fisher Scientific, Waltham, MA, USA), washed, incubated with HRP-conjugated secondary goat anti-mouse IgG (1:5000, A28177, Thermo Fisher Scientific, Waltham, MA, USA), and developed using Chemiluminescent Super Signal West Pico Substrate (Thermo Fisher Scientific, Waltham, MA, USA). After stripping, blots were incubated with anti-Lamin B1 rabbit polyclonal antibody (1:5000, PA5-19468, Thermo Fisher Scientific, Waltham, MA, USA), then washed, incubated with HRP-conjugated secondary goat anti-rabbit IgG (1:8000, A0545, Sigma-Aldrich, Steinheim, Germany), and developed as described above. The band intensities (55 kDa for FXR, 66 kDa for Lamin B1) were calculated as FXR/Lamin B1 ratio and presented as a percent of control.

### 4.6. Bile Acid Measurement

Total bile acid level was measured in plasma and brain tissue homogenates (cortex and cerebellum) using Bile Acid Fluorescence Kit (Sigma Aldrich, Steinheim, Germany) according to the manufacturer protocol.

### 4.7. Statistical Analysis

All experiments were carried out with replicates in terms of animals per group as indicated. The data are presented as the mean ± standard error of the mean (mean ± SEM). The results were analyzed by an unpaired Student’s t-test. *p*-values < 0.05 were considered statistically significant. All analyses were performed using Statistica for Windows v. 8.0 (Statsoft. Inc., Palo Alto, CA, USA).

## Abbreviations

ALF	Acute liver failure
BA	Bile acid
BBB	Blood–brain barrier
FXR	Farnesoid X receptor
HE	Hepatic encephalopathy
SHP	Small heterodimer partner
TAA	Thioacetamide

## Appendix A

**Table A1:** Plasma level of ammonia (NH_3_) and activities of aspartate aminotransferase (AST), alanine amino transferase (ALT), and γ-glutamyl transpeptidase (GGTP) in control and thioacetamide (TAA)-treated rats.

	Control	TAA
AST (GOT) (U/L)	153.84 ± 12.4	1001.82 ± 141.42 ***
ALT (GPT) (U/L)	60.18 ± 1.92	336.38 ± 53.75 ***
GGTP (U/L)	2.88 ± 0.15	4.1 ± 0.41 *
NH_3_ (µmol/L)	55.91 ± 4.52	120.85 ± 13.39 **

*** *p* < 0.001, ** *p* < 0.01, * *p* < 0.05 vs. control group, *n* = 5 in each group

## References

[1] Butterworth R.F. (2016). Pathogenesis of hepatic encephalopathy in cirrhosis: The concept of synergism revisited. Metab. Brain Dis..

[2] Shawcross D.L., Wright G., Olde Damink S.W.M., Jalan R. (2007). Role of ammonia and inflammation in minimal hepatic encephalopathy. Metab. Brain Dis..

[3] Lima L.C.D., Miranda A.S., Ferreira R.N., Rachid M.A., e Silva A.C.S. (2019). Hepatic encephalopathy: Lessons from preclinical studies. World J. Hepatol..

[4] Bron B., Waldram R., Silk D.B.A., Williams R. (1977). Serum, cerebrospinal fluid, and brain levels of bile acids in patients with fulminant hepatic failure. Gut.

[5] Yin P., Wan D., Zhao C., Chen J., Zhao X., Wang W., Lu X., Yang S., Gu J., Xu G. (2009). A metabonomic study of hepatitis B-induced liver cirrhosis and hepatocellular carcinoma by using RP-LC and HILIC coupled with mass spectrometry. Mol. Biosyst..

[6] Wang X., Xie G., Zhao A., Zheng X., Huang F., Wang Y., Yao C., Jia W., Liu P. (2016). Serum Bile Acids Are Associated with Pathological Progression of Hepatitis B-Induced Cirrhosis. J. Proteome Res..

[7] Xie G., Wang X., Huang F., Zhao A., Chen W., Yan J., Zhang Y., Lei S., Ge K., Zheng X. (2016). Dysregulated hepatic bile acids collaboratively promote liver carcinogenesis. Int. J. Cancer.

[8] McMillin M., Frampton G., Quinn M., Ashfaq S., Santos M.D.L., Grant S., Demorrow S. (2016). Bile Acid Signaling Is Involved in the Neurological Decline in a Murine Model of Acute Liver Failure. Am. J. Pathol..

[9] Hadjihambi A., Harrison I.F., Costas-Rodríguez M., Vanhaecke F., Arias N., Gallego-Durán R., Mastitskaya S., Hosford P.S., Olde Damink S.W.M., Davies N. (2019). Impaired brain glymphatic flow in experimental hepatic encephalopathy. J. Hepatol..

[10] Xie G., Wang X., Jiang R., Zhao A., Yan J., Zheng X., Huang F., Liu X., Panee J., Rajani C. (2018). Dysregulated bile acid signaling contributes to the neurological impairment in murine models of acute and chronic liver failure. EBioMedicine.

[11] Quinn M., Mcmillin M., Galindo C., Frampton G., Yong H., Demorrow S. (2014). Bile acids permeabilize the blood brain barrier after bile duct ligation in rats via Rac1-dependent mechanisms. Dig. Liver Dis..

[12] Mertens K.L., Kalsbeek A., Soeters M.R., Eggink H.M. (2017). Bile Acid Signaling Pathways from the Enterohepatic Circulation to the Central Nervous System. Front. Neurosci..

[13] Keitel V., Org B.G., Bidmon H.J., Zemtsova I., Spomer L. (2010). The Bile Acid Receptor TGR5 (Gpbar-1) Acts as a Neurosteroid Receptor in Brain. Glia.

[14] Huang C., Wang J., Hu W., Wang C., Lu X., Tong L., Wu F. (2016). Identification of functional farnesoid X receptors in brain neurons. FEBS Lett..

[15] Lu T.T., Makishima M., Repa J.J., Schoonjans K., Kerr T.A., Auwerx J., Mangelsdorf D.J. (2000). Molecular Basis for Feedback Regulation of Bile Acid Synthesis by Nuclear Receptors. Mol. Cell.

[16] Massafra V., Milona A., Vos H.R., Ramos R.J.J., Gerrits J., Willemsen E.C.L., Pittol J.M.R., Ijssennagger N., Houweling M., Prinsen H.C.M.T. (2017). Farnesoid X Receptor Activation Promotes Hepatic Amino Acid Catabolism and Ammonium Clearance in Mice. Gastroenterology.

[17] McMillin M., Grant S., Frampton G., Petrescu A.D., Kain J., Williams E., Haines R., Canady L., Demorrow S. (2018). FXR-Mediated Cortical Cholesterol Accumulation Contributes to the Pathogenesis of Type A Hepatic Encephalopathy. Cell. Mol. Gastroenterol. Hepatol..

[18] Huang F., Wang T., Lan Y., Yang L., Pan W., Zhu Y. (2015). Deletion of mouse FXR gene disturbs multiple neurotransmitter systems and alters neurobehavior. Front. Behav. Neurosci..

[19] Rodrigo R., Cauli O., Gomez-Pinedo U., Agusti A., Hernandez-Rabaza V., Garcia-Verdugo J.M., Felipo V. (2010). Hyperammonemia induces neuroinflammation that contributes to cognitive impairment in rats with hepatic encephalopathy. Gastroenterology.

[20] Felipo V. (2013). Hepatic encephalopathy: Effects of liver failure on brain function. Nat. Rev. Neurosci.

[21] Balzano T., Forteza J., Molina P., Giner J., Monzó A., Sancho-Jiménez J., Urios A., Montoliu C., Felipo V. (2018). The Cerebellum of Patients with Steatohepatitis Shows Lymphocyte Infiltration, Microglial Activation and Loss of Purkinje and Granular Neurons. Sci. Rep..

[22] García-García R., Cruz-Gómez Á.J., Urios A., Mangas-Losada A., Forn C., Escudero-García D., Kosenko E., Torregrosa I., Tosca J., Giner-Durán R. (2018). Learning and Memory Impairments in Patients with Minimal Hepatic Encephalopathy are Associated with Structural and Functional Connectivity Alterations in Hippocampus. Sci. Rep..

[23] Zheng L., Yin L., Xu L., Qi Y., Li H., Xu Y., Han X., Liu K., Peng J. (2018). Protective effect of dioscin against thioacetamide-induced acute liver injury via FXR/AMPK signaling pathway in vivo. Biomed. Pharmacother..

[24] Keshk W.A., Soliman N.A., Ali D.A., Elseady W.S. (2019). Mechanistic evaluation of AMPK/SIRT1/FXR signaling axis, inflammation, and redox status in thioacetamide-induced liver cirrhosis: The role of *Cichorium intybus* linn (chicory)-supplemented diet. J. Food Biochem..

[25] Verbeke L., Mannaerts I., Schierwagen R., Govaere O., Klein S., Vander Elst I., Windmolders P., Farre R., Wenes M., Mazzone M. (2016). FXR agonist obeticholic acid reduces hepatic inflammation and fibrosis in a rat model of toxic cirrhosis. Sci. Rep..

[26] Gao X., Wang C., Ning C., Liu K., Wang X., Liu Z., Sun H., Ma X., Sun P., Meng Q. (2018). Hepatoprotection of auraptene from peels of citrus fruits against thioacetamide-induced hepatic fibrosis in mice by activating farnesoid X receptor. Food Funct..

[27] Zhao Q., Liu F., Cheng Y., Xiao X.-R., Hu D.-D., Tang Y.-M., Bao W.-M., Yang J.-H., Jiang T., Hu J.-P. (2019). Celastrol Protects From Cholestatic Liver Injury Through Modulation of SIRT1-FXR Signaling. Mol. Cell. Proteom..

[28] Butterworth R.F., Norenberg M.D., Felipo V., Ferenci P., Albrecht J., Blei A.T. (2009). Experimental models of hepatic encephalopathy: ISHEN guidelines. Liver Int..

[29] Jayakumar A.R., Ruiz-Cordero R., Tong X.Y., Norenberg M.D. (2013). Brain edema in acute liver failure: Role of neurosteroids. Arch. Biochem. Biophys..

[30] Grant S., McMillin M., Frampton G., Petrescu A.D., Williams E., Jaeger V., Kain J., DeMorrow S. (2018). Direct Comparison of the Thioacetamide and Azoxymethane Models of Type A Hepatic Encephalopathy in Mice. Gene Expr..

[31] Horvatits T., Drolz A., Roedl K., Rutter K., Ferlitsch A., Fauler G., Trauner M., Fuhrmann V. (2017). Serum bile acids as marker for acute decompensation and chronic liver failure in patients with non-cholestatic cirrhosis. Liver Int..

[32] Nguyen J.H., Yamamoto S., Steers J., Sevlever D., Lin W., Shimojima N., Castanedes-Casey M., Genco P., Golde T., Richelson E. (2006). Matrix metalloproteinase-9 contributes to brain extravasation and edema in fulminant hepatic failure mice. J. Hepatol..

[33] Shimojima N., Eckman C.B., McKinney M., Sevlever D., Yamamoto S., Lin W., Dickson D.W., Nguyen J.H. (2008). Altered expression of zonula occludens-2 precedes increased blood-brain barrier permeability in a murine model of fulminant hepatic failure. J. Invest. Surg..

[34] McMillin M.A., Frampton G.A., Seiwell A.P., Patel N.S., Jacobs A.N., DeMorrow S. (2015). TGFβ1 exacerbates blood–brain barrier permeability in a mouse model of hepatic encephalopathy via upregulation of MMP9 and downregulation of claudin-5. Lab. Investig..

[35] Obara-Michlewska M., Ding F., Popek M., Verkhratsky A., Nedergaard M., Zielinska M., Albrecht J. (2018). Interstitial ion homeostasis and acid-base balance are maintained in oedematous brain of mice with acute toxic liver failure. Neurochem. Int..

[36] Masago K., Kihara Y., Yanagida K., Hamano F., Nakagawa S., Niwa M., Shimizu T. (2018). Lysophosphatidic acid receptor, LPA6, regulates endothelial blood-brain barrier function: Implication for hepatic encephalopathy. Biochem. Biophys. Res. Commun..

[37] Albrecht J., Hilgier W., Januszewski S., Kapuściński A., Quack G. (1994). Increase of the brain uptake index for L-ornithine in rats with hepatic encephalopathy. Neuroreport.

[38] Albrecht J., Hilgier W., Januszewski S., Quack G. (1996). Contrasting effects of thioacetamide-induced liver damage on the brain uptake indices of ornithine, arginine and lysine: Modulation by treatment with ornithine aspartate. Metab. Brain Dis..

[39] Szumanska G., Albrecht J. (1997). Lectin histochemistry of the rat brain following thioacetamide-induced hepatic failure. Mol. Chem. Neuropathol..

[40] Jin S., Wang X.-T., Liu L., Yao D., Liu C., Zhang M., Guo H.-F., Liu X.-D. (2013). P-glycoprotein and multidrug resistance-associated protein 2 are oppositely altered in brain of rats with thioacetamide-induced acute liver failure. Liver Int..

[41] Balasubramaniyan N., Ananthanarayanan M., Suchy F.J. (2012). Direct methylation of FXR by Set7/9, a lysine methyltransferase, regulates the expression of FXR target genes. Am. J. Physiol. Gastrointest. Liver Physiol..

[42] Choi W.T., Tosun M., Jeong H.-H., Karakas C., Semerci F., Liu Z., Maletić-Savatić M. (2018). Metabolomics of mammalian brain reveals regional differences. BMC Syst. Biol..

[43] Tripodi V., Contin M., Fernández M.A., Lemberg A. (2012). Bile acids content in brain of common duct ligated rats. Ann. Hepatol.

[44] Fiorucci S., Rizzo G., Donini A., Distrutti E., Santucci L. (2007). Targeting farnesoid X receptor for liver and metabolic disorders. Trends Mol. Med..

[45] Verbeke L., Farre R., Trebicka J., Komuta M., Roskams T., Klein S., Elst I.V., Windmolders P., Vanuytsel T., Nevens F. (2013). Obeticholic Acid, a Farnesoid X Receptor Agonist, Improves Portal Hypertension by Two Distinct Pathways in Cirrhotic Rats. Hepatology.

[46] Beuers U., Trauner M., Jansen P., Poupon R. (2015). New paradigms in the treatment of hepatic cholestasis: From UDCA to FXR, PXR and beyond. J. Hepatol..

[47] Schousboe A., Drejer J., Hansen G.H., Meier E. (1985). Cultured Neurons as Model Systems for Biochemical and Pharmacological Studies on Receptors for Neurotransmitter Amino Acids. Dev. Neurosci..

[48] Zielińska M., Milewski K., Skowrońska M., Gajos A., Ziemińska E., Beręsewicz A., Albrecht J. (2015). Induction of inducible nitric oxide synthase expression in ammonia-exposed cultured astrocytes is coupled to increased arginine transport by upregulated y(+)LAT2 transporter. J. Neurochem..

[49] Skowrońska K., Obara-Michlewska M., Czarnecka A., Dąbrowska K., Zielińska M., Albrecht J. (2019). Persistent Overexposure to N-Methyl-d-Aspartate (NMDA) Calcium-Dependently Downregulates Glutamine Synthetase, Aquaporin 4, and Kir4.1 Channel in Mouse Cortical Astrocytes. Neurotox. Res..

[50] Livak K.J., Schmittgen T.D. (2001). Analysis of Relative Gene Expression Data Using Real-Time Quantitative PCR and the 2−ΔΔCT Method. Methods.

[51] Czarnecka A., Milewski K., Jaźwiec R., Zielińska M. (2017). Intracerebral Administration of S-Adenosylhomocysteine or S-Adenosylmethionine Attenuates the Increases in the Cortical Extracellular Levels of Dimethylarginines Without Affecting cGMP Level in Rats with Acute Liver Failure. Neurotox. Res..

